# Oxidized Cholesterol Derivatives in Fraction B Prepared from Gulf Catfish (*Arius bilineatus*, Val.) Skin Regulate Calcium Response and Neutrophil Extracellular Traps (NETs) Formation

**DOI:** 10.3390/biomedicines12071380

**Published:** 2024-06-21

**Authors:** Jassim M. Al-Hassan, Mohammad Afzal, Sosamma Oommen, Yuan Fang Liu, Meraj Khan, Cecil Pace-Asciak

**Affiliations:** 1 Biological Sciences Department, Faculty of Science, Kuwait University, Kuwait City 15462, Kuwait or jassimmalhassan@gmail.com (J.M.A.-H.); or afzalq8@gmail.com (M.A.); or jubintom@hotmail.com (S.O.); 2 Health Sciences Research Center, Khaldiya Campus, Abdullah Al-Salem University, Khaldiya, Kuwait City 72303, Kuwait; 3 Translational Medicine, Research Institute, The Hospital for Sick Children, Toronto, ON M5G OA4, Canada; liuyuanfanh@gmail.com (Y.F.L.); meraj.khan@sickkids.ca (M.K.); 4 Department of Pharmacology, Faculty of Medicine, University of Toronto, Toronto, ON M5S 1A8, Canada

**Keywords:** catfish, skin secretion, Fraction B, sterols/steroids, calcium release, formation of NETs

## Abstract

In this study, we present in vitro actions of pure commercial preparations of oxidized and/or dehydrated metabolites of cholesterol (OS) identified in the lipid fraction of Fraction B (FB) prepared from a catfish skin preparation on calcium transients and on the formation of human neutrophil extracellular traps (NETs). These investigations are part of an ongoing effort to understand the important roles these compounds play as components of FB when FB is applied to accelerate the healing of wounds and the healing of highly infected non-healing diabetic foot ulcers, without the use of antibiotics. Our aim was to determine potential therapeutic interventions for various disease states. Our results reveal interesting findings, demonstrating specific actions of the individual compounds. Compounds 7α-hydroxy-cholesterol (S3), Cholestane-3,5,6-triol (S5), 5-cholesten-3β-ol-7-one (S8) and Cholesta-3,5 dien-7-one (S10) are inhibitory, while Cholesterol 5β,6β-epoxide (S4) and 5α-cholestane-3,6-dione (S11) activate the response for calcium influx in human neutrophils. A somewhat similar response is observed in dHL60 cell lines, where S3, S5, S7, S8, and cholesta-2,4-diene (S14) inhibit the calcium influx, although S4, S10, and S11 activate the response in this cell line. Furthermore, we observed a relationship between actions against NETosis and calcium transients. Interestingly, relative to the vehicle control, S3, Cholesta-3,5 diene (S9), and S14 appeared to significantly stimulate DNA release (NETosis), while S2, 7α-hydroxy-cholesterol (S6) and cholesta-3,5 dien-7-one (S10) caused lesser stimulation. We provide the IC50 activities for each compound tested in each assay. Calcium influx and NETs formation (NETosis) correlate with diseases exacerbation. These findings offer valuable insights into the potential therapeutic applications of individual OS for various diseases, highlighting their importance in future interventions.

## 1. Introduction

The identification of medicinally useful products from nature has been important in drug discovery, serving as the basis for the control/treatment of many diseases/conditions, including cancer, inflammation, and diabetes, and serves as a basis for the further chemical development of more therapeutically useful and more stabilized compounds [1,2].

Due to the complexity in the isolation of individual compounds in nature, despite modern technology and because most biologically active substances of interest occur in minor amounts, the route preferred for the detection of important bioactive compounds that are useful in treating disease is to use extracts from nature to investigate them for the presence of biologically active compounds. These compounds should relate to a specific disease/condition, i.e., contraction or relaxation of blood vessels for hypertension, blood glucose lowering effects for diabetes, inflammatory cytokines for inflammatory disease, anti-proliferation for cancer etc. It is far simpler to identify the presence of biological actions in crude extracts from nature (plants and marine organisms) and to then follow up with semi-purified extracts of interest, with subsequent identification of compounds of interest through conventional chromatographic means and mass spectrometry. It is beneficial when identified compounds of a known structure are available commercially. Similarly, it is beneficial to test these compounds in order to explore new potential uses through bioassays that relate to the in vitro assays of diseases or via in animal disease models in vivo.

The present study is part of an interesting utilization of the above process, with an ongoing investigation initiated by one of the authors (J.M.A.-H.) decades ago that seeks to investigate the properties of an epidermally released gel, first in its crude form and then after partial purification in order to identify further actions and even to identify individual proteins and lipids within this gel [3]. The semi purified gel and fractions within it has been shown to possess platelet aggregating substances, pain suppressing actions, and wound healing properties in people and was seen to repair damaged blood vessels in rodents [4,5,6,7].

The semi purified preparation containing lipids and proteins, named Fraction B (FB) was shown to accelerate wound healing and healing of highly infected diabetic foot ulcers, control the growth of pancreatic cancer [8] and to effect regeneration of injured sciatic nerves [7]. Within the lipid fraction, we have demonstrated the presence of a cholesterol metabolite (previously named S-5 viz. Cholesterol-3,5-diene) and a furanoic fatty acid, (named F-6, viz. [12,15-epoxy-13,14-dimethyleicosa-12,14-dienoic acid]) that suppressed proliferation of human cancer cells in vitro [9].

We have recently shown that, of the 14 OS identified in FB [10], one OS (S5) was specifically active in suppressing cancer cell proliferation of the leukemic cell line K-562, although it was less active than other OS on breast cancer cell lines. Neutrophil extracellular traps (NETs) are formed following the activation of neutrophils and play an important role in the development of cancer, especially metastatic disease [11].

The aim of this study was to compare the biological actions of all 14 OS on various in vitro bioassays ongoing in The Hospital for Sick Children, Toronto (C.P.-A., SickKids lab., Toronto, ON, Canada) to investigate whether a structural relationship exists between the diverse compounds of interest and their various observed biological activities which may be useful in suggesting structural modifications for therapeutic development.

## 2. Materials and Methods

All OS used in these studies were identified previously from the gel released from the skin of caught catfish and for this in vitro study were purchased from Sigma-Aldrich, (Oakville, ON, Canada) and Steraloids (Newport, RI, USA). RPMI 1640, fetal bovine serum (FBS), antibiotics (penicillin and streptomycin), phosphate buffered saline (PBS) trypan blue and trypsin-ethylenediamine tetra acetic acid (trypsin-EDTAz) were purchased from Wisent Inc. (St. Bruno, QC, Canada), while SFM PMI medium was acquired from Thermo Fisher Scientific, Waltham, MA, USA.

### 2.1. Isolation of Human Peripheral Neutrophils

Peripheral blood from healthy donors was collected in K2 EDTA blood collection tubes (BD, Franklin Lakes, NJ, USA). Signed informed consent was obtained from each donor, and the protocol was approved by the Hospital for Sick Children ethics committee (REB#100049654). Neutrophils were then isolated from whole blood using PolymorphPrep (Axis-Shield PoC, Oslo, Norway) according to the manufacturer’s instructions with minor modifications and as reported previously by our lab [12]. The modifications are as follows: (i) the lysis of red blood cells was undertaken using a hypotonic saline solution (0.2% (*w*/*v*) NaCl) and was followed by the addition of an equal volume of 1.6% (*w*/*v*) NaCl solution with HEPES buffer (20 mM, pH 7.2) to make the solution buffered and isotonic. This was followed by two washes with a wash buffer containing 0.85% (*w*/*v*) NaCl and HEPES (10 mM, pH 7.2). Isolated neutrophils were then resuspended in RPMI (Invitrogen) supplemented with HEPES buffer (10 mM, pH 7.2). The isolated neutrophils used in the experiments were >95% live and pure.

### 2.2. HL60 Cell Line Differentiation into dHL60

HL-60 cells (CCL-240, ATCC, Manassas, VA, USA) were differentiated into a neutrophil-like state by treatment with DMSO (1.25%) for three to six days. Cell culture was undertaken with RPMI 1640, 10% heat-inactivated fetal bovine serum (FBS), and 1% penicillin/streptomycin antibiotics at 37 °C in 5% CO_2_. Cells were passaged two to three times per week, maintaining cell densities between 1–2 × 10^6^ cells/mL in cell culture flasks. On day six, we washed the cells and used them for further experimentations.

### 2.3. Measurement of Intracellular Calcium ([Ca^2+^]i)

We used a method to measure intracellular calcium concentrations that was previously described with human neutrophils. Briefly, neutrophils were incubated with 3 mM (final concentration 3 µM) of the acetoxymethyl ester precursor of the calcium indicator indo-1 over 45 min at 37 °C. The supernatant containing excess dye was removed and the dye-loaded neutrophil pellet was gently resuspended in the same fresh medium (containing no CaCl_2_). Aliquots of 2 × 10^6^ cells were added to 1 mL indicated medium in a quartz cuvette (Diamed Lab., Toronto, ON, Canada) and equilibrated with 1 mM CaCl_2_ or EGTA. The cell suspension was continuously stirred magnetically, and the temperature was controlled at 37 °C. Intracellular calcium concentrations were monitored with a PerkinElmer fluorescence spectrophotometer (model 650-40) and recorded on a chart recorder (LKB model 2210) set at 1 cm/min. The excitation wavelength was set at 335 nm and the emission wavelength at 405 nm, with slits of excitation and emission set at 3 and 15 nm, respectively. Each sample was stirred for 1 min in the spectrofluorometer before any addition. Typical measurement was initiated by addition of 1 µL of glass distilled ethanol or test compound in ethanol followed 2 min later by fMLP (7.5 mM). The resulting effect was recorded for the next 6 min. At the end of the test, a calibration was carried out to determine the maximal fluorescence by adding ionomycin at 1 mM (final concentration 1 µM) and minimal fluorescence by adding MnCl_2_ (final concentration 3 mM).

### 2.4. NETosis Assay

NETosis kinetics (%DNA release) were evaluated using Sytox Green, a cell-impermeable DNA binding dye. The Sytox Green dye demonstrates a >500-fold fluorescence enhancement upon binding with nucleic acids. Hence, the fluorescence emitted by the dye serves as a measure of DNA release. A suspension of 50,000 cells in 50 µL volume, along with 5 µM Sytox Green dye, was seeded into 96-well plates. Following the addition of different compounds (S1 to S14) and cells activation, changes in fluorescence intensities were measured every 30 min for up to 210 min using a POLARstar OMEGA (BMG Labtech, Ortenberg, Germany) with excitation at 504 nm and emission at 523 nm. To calculate the extent of NETosis represented by percent DNA release, the fluorescence reading at time zero was subtracted from the fluorescence reading at each time point. This value was then divided by the total DNA amount, determined by the fluorescence values of neutrophils lysed with 0.5% (*w*/*v*) Triton X-100. Assays were excluded if the baseline activation exceeded 30%.

### 2.5. Statistical Analysis

Raw data compilation and normalization were calculated in Excel, whereas statistical analysis and graph generation were achieved using the GraphPad Prism software (Version 5.0a). The Student’s *t* test for comparing two groups and analysis of variance (ANOVA) with Bonferroni’s post-test or Dunnett’s test for more than two groups were used where appropriate. All data are presented as mean *±* standard error from the mean (SEM). A *p* value of <0.05 was considered statistically significant. The biological replicates and applied statistics are mentioned in each of the figure legends.

## 3. Results

### 3.1. Calcium Transients

**Figure 1:**
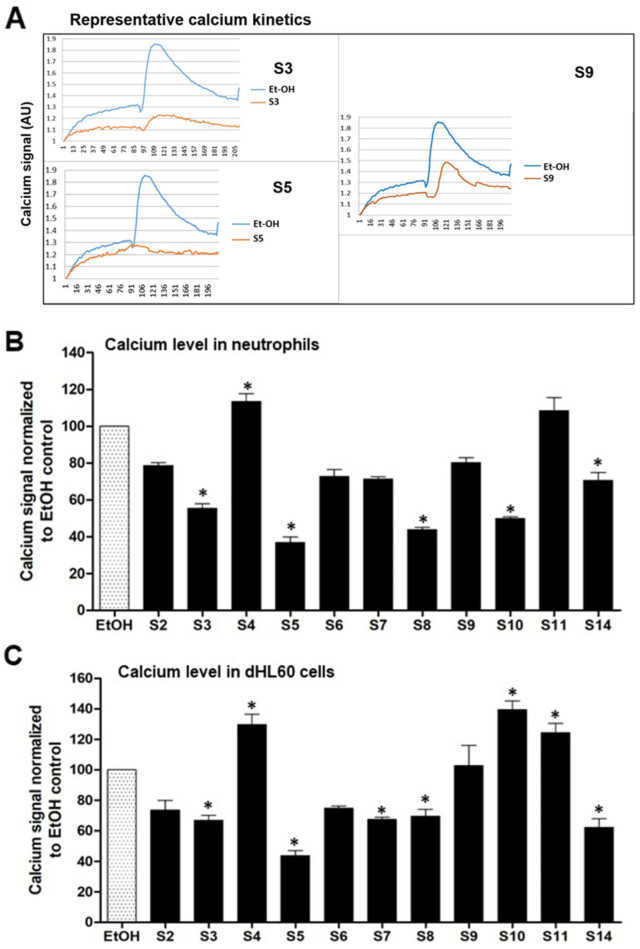
(**A**) Representative scans of fMLP-evoked calcium release by isolated neutrophils showing the kinetics of calcium release by three of the OS family described herein. Note the potent inhibition of the fMLP-evoked calcium release by S-5. (**B**) Quantitative data by various OS on fMLP-evoked calcium release in human neutrophils. (**C**) fMLP-evoked release in dHL60 cells in vitro. The effect of fMLP in the ethanol vehicle is shown and expressed as 100%. The values were normalized to the negative control values of the same experiment * indicates *p* value  < 0.05; *n* = 3, one-sample *t* test compared with hypothetical value 100 (normalized value).

Screening the 14 OS for calcium transients in both human neutrophils and dHL60 cells showed neutrophils to be more responsive to stimulation by fMLP. Figure 1A shows some typical scans of the kinds of responses (representative), where S3 and S5 show the inhibition of calcium influx near to base line in reference to control. Figure 1B compares the responses of the tested OS. Relative to the vehicle plus fMLP response (100%), several of the OS inhibited the response in human neutrophils while a number of compounds increased the calcium response. Among the most active inhibitors were S5, S8 and S10, as shown in Figure 1B, while S3 also shows inhibitory action. Activators of the response were S4 and S11. Somewhat similar responses in dHL60 cell lines (in vitro) are shown in Figure 1C where S3, S5, S7, S8 and S14 inhibited the fMLP response, though S4, S10 and S11 activated the response in this cell line.

### 3.2. NETosis

Compounds that alter calcium levels could regulate NETosis. Hence, we examined the roles of OS in NETosis. The percentage of DNA release in each condition is presented as a percentage of the total DNA within that experimental condition. This normalization helps in comparing the formation of NETs across different experiments and conditions. This method of presenting DNA release is well accepted and widely used.

**Figure 2:**
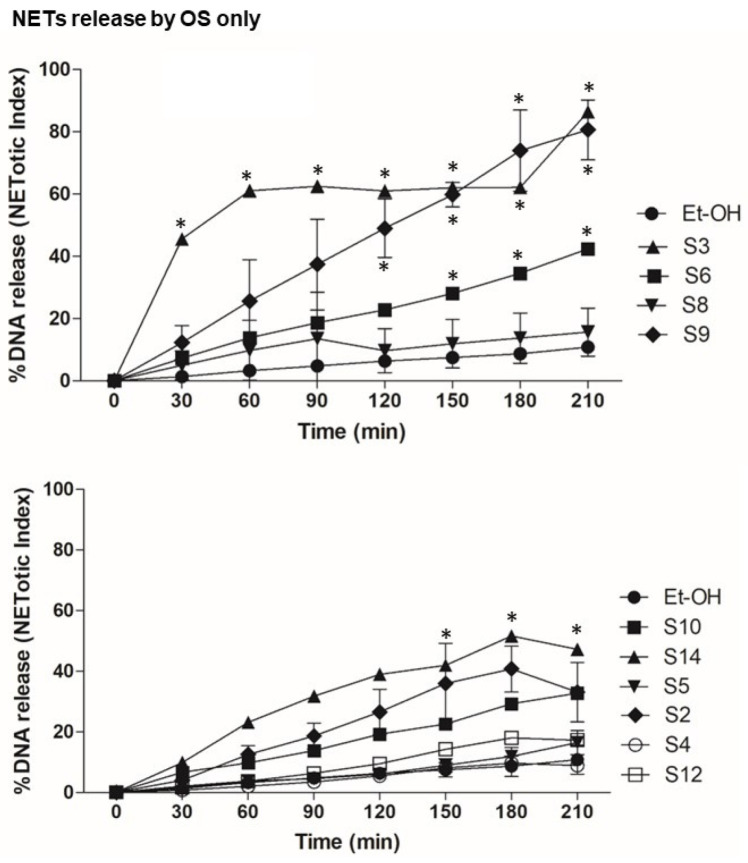
NETosis (%DNA release) was assessed using Sytox Green assay. Neutrophils were left unstimulated (Et-OH) in order to obtain baseline NETosis information and were treated with compounds only. The percentage of DNA release for each condition compared with Triton X-100 sample (100% DNA release) was calculated. These graphs show the NETosis by compounds only, in order to show the effect of compounds in neutrophils for NETosis. *n* = 3, * *p* < 0.05 between control and different stimulations, two-way ANOVA with Bonferroni’s multiple comparison post-test.

**Figure 3:**
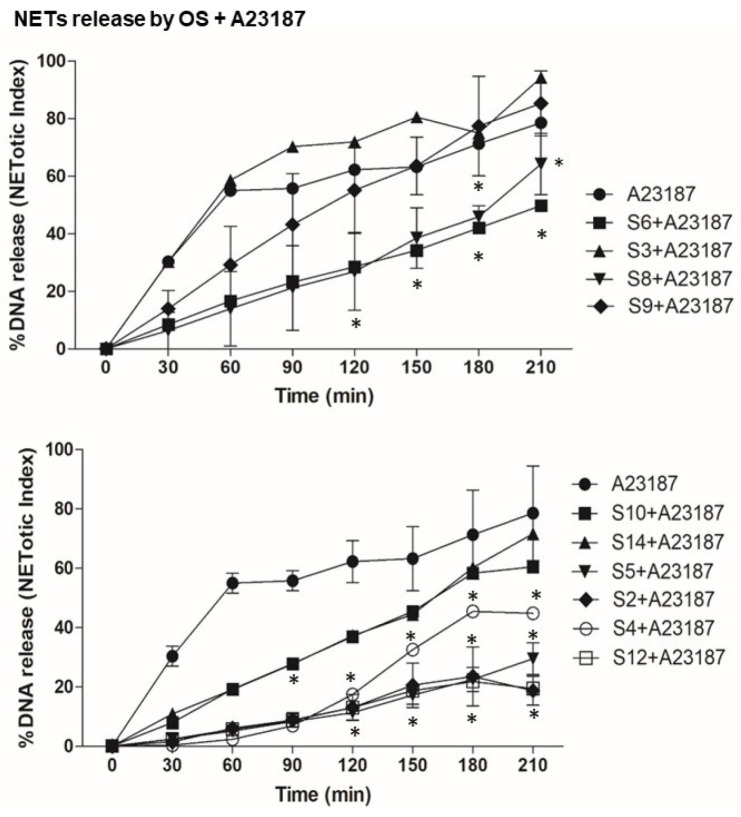
Neutrophils were stimulated by A23187 (calcium ionophore) alone in order to obtain nox-independent NETosis and treated with compounds in the presence of a stimulator (A23187). Graphs show the kinetics of %DNA release with only A23187—a nox-independent NETosis inducer and compounds. These kinetics graphs show the effect of compounds in the presence of a nox-independent stimulator. *n* = 3, * *p* < 0.05 between control and different stimulations, two-way ANOVA with Bonferroni’s multiple comparison post-test.

**Figure 4:**
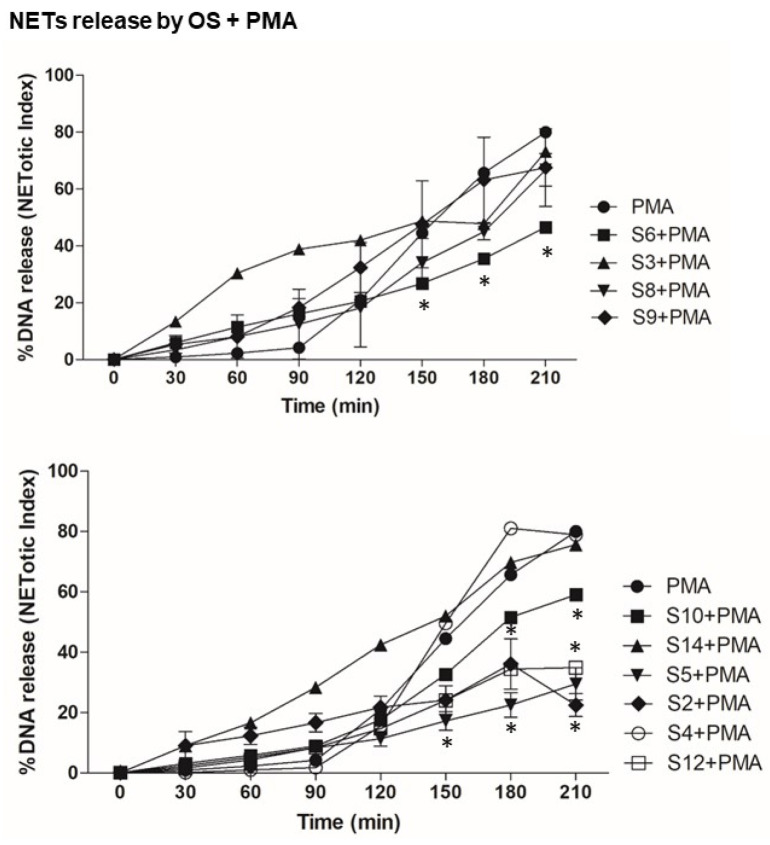
Neutrophils were stimulated by PMA (a known pharmacological NETosis inducer) alone to obtain nox-dependent NETosis and were treated with compounds in the presence of a stimulator (PMA). Graphs show the kinetics of %DNA release with only PMA—nox-independent NETosis inducer and compounds. These kinetics graphs show the effect of compounds in the presence of a nox-dependent stimulator. *n* = 3, * *p* < 0.05 between control and different stimulations, two-way ANOVA with Bonferroni’s multiple comparison post-test.

**Figure 5:**
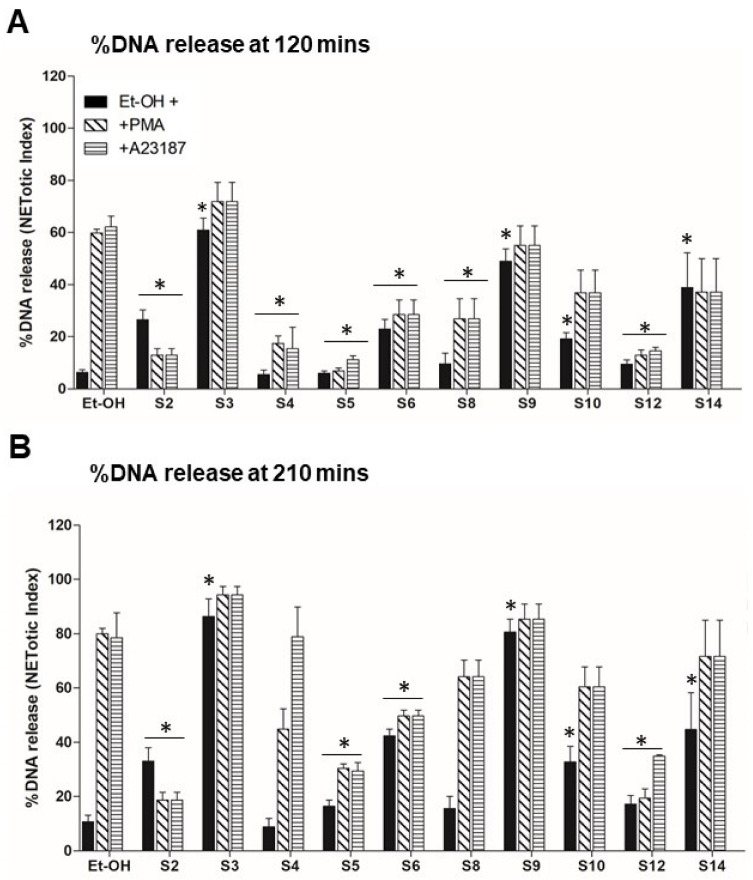
Bar diagram represents the %DNA release (NETosis), comparing the various OS tested at two time points without stimulation (black bar) and with stimulation with A23187 and PMA (cross hatched bars as per legend. Note the effectiveness of some OS at increasing activity at an early time point (120 min) and the increased distinction at 210 min (see specifically S-2, S-3, S-6, S-9, S-10, and S-14 increasing their activity in the absence of stimulation (black columns), and S-2, S-4, S-5, S-6, S-8, S-12 (inhibition of PMA and A23187 stimulation at 120 min). *n* = 3, * *p* < 0.05, one-way ANOVA with Tukey’s post-test compared with no drug control.

First, we tested the effect of OS alone on NETosis, as shown Figure 2. The NETosis kinetics and %DNA release shows that compounds S3, S6 and S9 themselves activate neutrophils for NETosis. To understand the effect of OS on the nox-independent pathway, we used OS and A23187 in the assay (Figure 3). OS, such as that of S2, S5 and S6, suppresses the NETosis, whereas that of S3, S4, and S9 shows no effect. To further understand the effect of OS on nox-dependent NETosis, we used OS and PMA in the same NETosis assay (Figure 4). Interestingly, S2, S5, and S12 suppress the PMA-mediated NETosis, while S3, S4 and S6 do not have a significant effect. Our findings are summarized in Figure 5, where the %DNA release (NETosis) has been represented at two different time points. Overall, relative to vehicle control, S3, S6, S9, and S14 appeared to greatly stimulate DNA release, while S2 and S10 were seen to cause lesser stimulation. It is noteworthy that S5, the compound that causes potent killing of the leukemic cancer cells, was not as active as other OS on NETosis on its own. However, it was quite potent in suppressing stimulation by both A23187 and PMA (hatched bars). Other inhibitors (even less potent) were S2, S6 and S12, especially at 210 min.

## 4. Discussion

Neutrophils play a vital role in host defense. When activated by proinflammatory signals, they migrate to injury sites or areas with localized viruses and bacteria. Here, they release granules to deactivate pathogens and employ neutrophil extracellular traps (NETs) as part of the defense mechanism. NETosis, the process involving neutrophil activation and the formation of DNA-based weblike NET structures, ensnares and eliminates invading pathogens. This cellular mechanism is extensively reviewed in [13]. NETs consist of decondensed DNA structures with a weblike morphology, adorned with nuclear proteins, and calcium’s role is crucial in facilitating NETosis.

While the term “NETosis” is commonly used to define the entire process of NET formation, its appropriateness has been questioned considering the findings that suggest different forms of NET formation [14]. This represents not only an important shift in terminology but also a significant challenge, as some groups, such as Yipp et al., have coined terms like “vital NETosis” to describe their work [15]. Consequently, other groups citing this work have continued to use this term.

The induction of NETosis by calcium ionophores was first reported by Neeli and Radic [16], who investigated the role of various agonists in activating peptidyl arginine deiminase 4 (PAD4). PAD4, present in large amounts in the cytosol, translocates to the neutrophil nucleus when bound to calcium facilitated by ionophores. There, it deiminates histone arginine residues into neutral citrulline, resulting in chromatin decondensation necessary for nox-independent NET formation. We observed hypercitrullination of histone H3 in NET formation mediated by nox-independent agonists but not by nox-dependent agonists, confirming the importance of histone citrullination in NET formation. This is further supported by findings that A23187, a nox-independent NET formation agonist, induces histone H3 citrullination, while PMA, a nox-dependent agonist, does not. Citrullination of promoters is crucial as it provides access to transcription factors necessary for NET formation [17].

In this study, we have conducted experiments to examine the effects of commercially available compounds representing OS compounds identified from the skin secretions of the catfish, *Arius bilineatus*, specifically those fish collected from the Arabian Gulf [18]. We observed distinct patterns of biological activity associated with these OS fractions when tested in vitro for their impact on calcium transients induced by fMLP and on NETosis triggered by PMA or A23187. In a prior study [10], we identified one particular sterol in the OS fraction, Cholestane-3,5,6-triol (newly referred to as S5 in Table 1), which exhibited unique and potent cytotoxic effects against human cancer cells in vitro. Notably, it displayed a high degree of specificity for the leukemic cell line K562 and showed somewhat lesser activity against two breast cancer cell lines [10].

Heightened levels of intracellular calcium, whether prompted by the liberation of calcium from endoplasmic reticulum (ER) reservoirs or via calcium channels in the plasma membrane, play a pivotal role in instigating NETosis. As substantiated by Wang et al. in a 2009 study, this physiological phenomenon is prominently evoked by inflammatory agents such as LPS, PMA, IL-8, and A23187 [19]. Conversely, the inhibition of NETosis can be accomplished through the concurrent chelation of intracellular calcium release and the blockade of extracellular calcium influx [20].

The study’s examination of individual structures of the OS compounds yields noteworthy findings. (i) Concerning the crucial role of calcium in NETs activation, two compounds, S8 and S10, appear to inhibit calcium activation by fMLP (Figure 1); structurally, both compounds exhibit conjugation between the carbon 3–7 region. Specifically, S8 features a 3-keto-4,5 double bond, while S10 possesses a keto group at C7 and an extended conjugated double bond at C3,4 and C5,6 (see Table 1). (ii) When these two compounds are administered individually (represented by solid black bars in Figure 5), they do not have a significant impact on NETosis (DNA release). This lack of effect may be because they inhibit intracellular calcium rather than promoting the increase required for stimulating NETosis. (iii) S9 and S11 appear to stimulate the fMLP response (as shown in Figure 1) and correspondingly activate the release of DNA (NETosis) when administered in the absence of agonist stimulation (Figure 5).

In the present study, we observed the specific effects of two sterols, namely S8 and S10, from the OS fractions, inhibiting fMLP-induced calcium transients in human neutrophils. There was a lesser specificity in the dHL60 cell line (a leukemic promyelocytic cell line differentiated into neutrophils). Interestingly, S11 and S4 had a partially stimulating effect on fMLP-induced calcium transients. Furthermore, we investigated the impact of various OS compounds on NETosis. Some of these compounds, including S2, S3, S6, S7, S9, S10, and S-14 (represented by black bars), exhibited significant stimulation of NETosis when assessed on their own in the absence of agonist stimulation (as compared with a control vehicle). However, when evaluating the specific responses to agonist induced NETosis, we observed a less uniform pattern of action among the OS compounds. For instance, S2, S4, S5, S6, and S12 inhibited both PMA and A23187-induced NETosis (as indicated by hatched bars), suggesting that these OS compounds had the ability to suppress the stimulated inflammatory response in human neutrophils. These findings shed light on the nuanced and context-dependent effects of these OS compounds on calcium transients and NETosis in human neutrophils.

Because the various OS in their structural and biological diversity occur in the same preparation (FB, a semi-purified fraction of the catfish skin gel that contains a variety of proteins and lipids, that in turn contain OS and F-acids) their efficacy in various disease/conditions would benefit by their selection for different biological needs. Clearly, a mixture of OS as shown in this study and previous studies [6,8,10] with specific biological effects on various cell lines could reflect their effects in various conditions (inflammation, cancer, platelet aggregation etc.) and will be important to evaluate as predictors for therapeutic intervention. In this respect Al-Hassan has had a significant impact in showing the effectiveness of preparations of FB when treating diverse human conditions, including neurological pain and chronic demyelinated polyneuropathy, and in the treatment of highly infected diabetic foot ulcers with osteomyelitis without the use of antibiotics [18,21], the healing of injured skin and broken bones resulting from accidents, and the healing of some dermatological problems in humans. All cases were treated topically with FB or derivatives therefrom. Preliminary results have shown regeneration of some organs and tissues affected by diabetes in animals by application of FB (private communication).

Table 1 presents the IC50 values of the aforementioned OS compounds as evaluated in two distinct assays: firstly, using the leukemic cell line K-562, sourced from a prior publication for purposes of comparison [10]; and secondly, measuring calcium release from the dHL60 cell line.

The present naming of S5 in this paper reflects the naming of one compound in the family of OS identified and investigated in our recently published paper [10] (i.e., Cholestane- 3,5,6-triol) and NOT as referred to in the earlier published manuscript [6] as Cholesta-3,5-diene. As indicated in Table 1 of the present paper, Cholesta-3,5-diene is now represented as S9.

We suggest that a semi-pure preparation, as in the previously described FB fraction by Al Hassan, with all its diversity (protein growth factors [5] and lipid anti-inflammatory compounds [6,8,9,10] would offer a wider beneficial value than single purified compounds for the therapeutic management of disease. The FB preparation in its diversity of material has shown safety [7], effectiveness [8] and utility in several unrelated disease indications [5,7].

## Figures and Tables

**Table 1:** The IC50 of various OS used in different assays.

Number	Steroid/OS Used	MW	IC50 (µM)	
			K562	dHL-60
S1	Cholesterol	386.67	2.59(+)	>30.00
S2	7α-hydroxy-cholesterol	402.65	9.19	24.84
S3	7β-hydroxy cholesterol	402.65	7.45	17.38
S4	Cholesterol 5β,6β-epoxide	402.65	>100	>30.00
S5	Cholestane-3,5,6-triol	427.71	8.80	23.77
S6	5-cholesten-3b-ol-7-one	400.64	50.00	>30.00
S7	Progesterone	314.46	12.72(+)	>30.00
S8	(+)-4-cholestene-3-one	384.64	15.6	10.4
S9	Cholesta-3,5 diene	368.65	14.82	0.30
S10	Cholesta-3,5 dien-7-one	382.64	10.45	6.8(+)
S11	5α-cholestane-3,6-dione	400.65	6.24(+)	>30.00
S12	4, 6-cholestadien-3-one	382.62	10.45	37.90
S14	Cholesta-2,4-diene	368.65	15.00	2.71

(+) refers to stimulation.

## Data Availability

Data is contained within the article.
